# The Diagnostic Value of the Interstitial Biomarkers KL-6 and SP-D for the Degree of Fibrosis in Combined Pulmonary Fibrosis and Emphysema

**DOI:** 10.1155/2012/492960

**Published:** 2012-02-28

**Authors:** Shigeki Chiba, Hiromitsu Ohta, Kyoko Abe, Shu Hisata, Shinya Ohkouchi, Yasushi Hoshikawa, Takashi Kondo, Masahito Ebina

**Affiliations:** ^1^Department of Pulmonary Medicine, Tohoku University Graduate School of Medicine, Sendai 980-8575, Japan; ^2^Department of Thoracic Surgery, Institute of Development, Aging and Cancer, Tohoku University, Sendai 980-8575, Japan

## Abstract

The combined pulmonary fibrosis and emphysema (CPFE) was reported first in 1990, but it has been comparatively underestimated until recently. Although the diagnostic findings of both emphysematous and fibrotic regions are detectable by high-resolution computed tomography (HRCT) of the chest, the degree of progressive fibrosis, which increases with emphysematous lesions, is difficult to evaluate. In this study, we hypothesized that the biomarkers for pulmonary fibrosis, surfactant protein D (SP-D), and KL-6 would serve as good indicators of fibrotic lesions in CPFE. We recruited 46 patients who had been diagnosed in our hospital with both emphysema and fibrosis by their CT scan image from April 2003 to March 2008. The correlation among their pulmonary function tests, composite physiologic index (CPI), and the serum levels of SP-D and KL-6 was evaluated. We found a correlation between KL-6 and %VC, %TLC, or CPI and between SP-D and %VC or CPI. Interestingly, the combined product of KL-6 and SP-D (KL-6xSP-D) was found to highly correlate with %VC and %TLC or CPI. These results show that both KL-6 and SP-D, and especially the product of SP-D and KL-6, are good indicators of the presence of fibrotic lesions in the lungs of CPFE patients.

## 1. Introduction

Since the combined pulmonary fibrosis and emphysema was first reported as “combined cryptogenic fibrosing alveolitis and emphysema” by Wiggins et al. in 1990 [1], the disease has come to be generally recognized worldwide and was listed as “an atypical phenotype of idiopathic interstitial pneumonia” in the 3rd version of the Clinical Guideline for Idiopathic Interstitial Pneumonia, published in 1991 by the Diffuse Pulmonary Disease Group of the Ministry of Health, Labour and Welfare of the Japanese Government. However, the disease was underidentified until 2005, when Cottin et al. reported a retrospective study of 61 patients with both emphysema and pulmonary fibrosis and described the syndrome comprehensively [2]. The diagnosis is based on high-resolution computed tomography (HRCT) of the chest, with findings that indicate emphysema of the upper lobe and pulmonary fibrosis of the lower lobe. CPFE is characterized by a relatively well-preserved lung volume, severely impaired carbon monoxide transfer, significant decrease in PaO_2_ on exercise, and a high prevalence of pulmonary hypertension leading to a poor prognosis [3, 4]. We expect that the extent of pulmonary fibrosis in CPFE patients might influence the outcome, because patients with CPFE have a worse prognosis than patients with emphysema [5]. However, it is difficult to evaluate the extent of pulmonary fibrosis in patients with CPFE. Vital capacity and DLco are closely influenced by emphysematous change. Wells et al. proposed the composite physiologic index (CPI), calculated from the individual predicted percentage values for VC, DLco, and FEV_1.0_ as a new indicator of the extent of pulmonary fibrosis, and this index was shown to represent the extent of pulmonary fibrosis on HRCT, adjusting for emphysema [6]. 

KL-6/MUC-1 (KL-6) and surfactant protein D (SP-D) are useful biomarkers in the diagnosis of various types of interstitial lung disease (ILD), including IPF [7]. KL-6 is a circulating high-molecular glycoprotein, classified as human MUC-1 [8, 9]. KL-6 is expressed on the surface membrane of alveolar epithelial cells and bronchiolar epithelial cells. SP-D belongs to the subgroup of the C-type lectin superfamily. It is secreted from Clara cells and alveolar epithelial cells. These biomarkers are reportedly elevated in IPF patients and related to the severity of pulmonary fibrosis [10]. In this context, we expected that KL-6 and SP-D would prove to be useful biomarkers for the evaluation of the extent of pulmonary fibrosis in patients with CPFE.

## 2. Patients and Methods

### 2.1. Patients Examined

We retrospectively analyzed 46 patients with CPFE who had been diagnosed as having both emphysema and fibrosis on their HRCT images in order to evaluate the correlation between the serum biomarkers (KL-6, SP-D) and pulmonary function indices, or CPI, which represents pulmonary fibrosis in patients with CPFE. The patients examined were obtained from the clinical data recorded at Tohoku University Hospital from April 2003 to March 2008. Exclusion criteria included the presence of other interstitial lung disease, including drug-induced interstitial lung disease, hypersensitivity pneumonitis, pneumoconiosis, sarcoidosis, pulmonary histiocytosis, lymphangioleiomyomatosis, and eosinophilic pneumonia, or other obvious causes.

### 2.2. Radiological Assessment

High-resolution computed tomography (HRCT) scans of the chest were reviewed independently by five pulmonologists (SC, HO, SH, SO, and ME). Cases were deemed acceptable for inclusion if the following criteria were met: (1) presence of emphysema on CT scan defined as well-demarcated areas of decreased attenuation in comparison with contiguous normal lung and characterized by a very thin (1 mm) or no margin, and/or multiple bullae (1 cm) with upper zone predominance; (2) presence of a diffuse parenchymal lung disease with significant pulmonary fibrosis on HRCT scan, defined as reticular opacities with peripheral and basal predominance, honeycombing, architectural distortion, and/or traction broncho-bronchiolo-ectasis; focal ground-glass opacities and/or areas of alveolar condensation may be present but should not be prominent. No patient had atelectasis secondary to a central lung cancer at the time of the initial diagnosis.

### 2.3. Clinical Assessment

Medical records were analyzed retrospectively. The data which was extracted included age, gender, smoking history, laboratory results, pulmonary function tests, PaO_2_, and PaCO_2_ at rest. Access to patients' medical records was approved by the Tohoku University School of Medicine, and patient confidentiality was carefully maintained.

### 2.4. Measurements

The serum levels of KL-6 and SP-D were determined with commercially available ELISA kits. We defined a high KL-6 value as being over 500 U/mL, and a high SP-D value as over 110 ng/mL. The composite physiologic index (CPI) was calculated as follows [6]:

(1) $$\begin{array}{cc} \text{CPI} & =91.0-0.65\times \text{\%DLco}-0.53\times \text{\%VC}\\ & \;+\;0.34\times \text{\%}{\text{FEV}}_{\text{1.0}}. \end{array}\;$$

### 2.5. Statistical Analysis

Univariate correlations between variables were compared using Spearman's rank correlation coefficient. Comparisons between groups were performed using Student's unpaired  *t*-test. All tests were two sided and performed at the 0.05 significance level. All data are expressed as the means ± SD.

## 3. Results

### 3.1. Imaging Characteristics of the CPFE Patients

**Figure 1:**
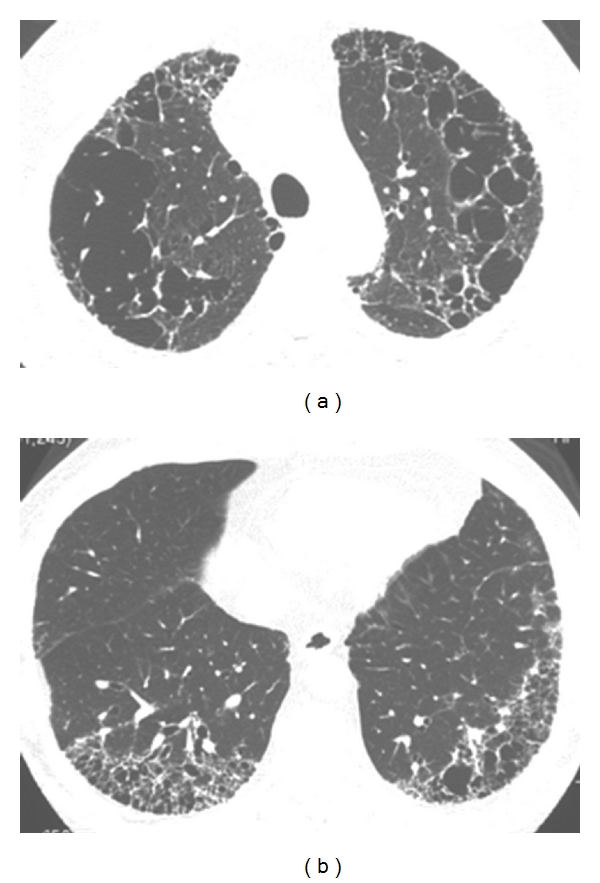
Imaging from a 64-year-old man with CPFE. (a) HRCT of bilateral upper lung fields shows emphysema. (b) HRCT of bilateral lower lung fields shows traction bronchiectasis and honeycomb and reticular opacities.

The diagnosis of CPFE was determined by HRCT scan which indicated the chief distribution of emphysema in the upper zones and pulmonary fibrosis in the lower zones of lungs. The prevalence of honeycombing, ground-glass opacities, and reticular opacities was particularly high. Typical HRCT scan images of these CPFE patients are shown in Figure 1.

### 3.2. Clinical Presentation and Pulmonary Function Test in CPFE

The clinical status and laboratory findings of the patients at the time of the initial diagnosis are presented in Table 1. Five patients had histologically diagnosed IPF/UIP. All patients had a history of smoking, and the current and former smokers made up 20 (43.5%) and 26 (56.5%) of the total, respectively. The partial pressure of oxygen in the arterial blood at rest and the partial pressure of carbon dioxide in the arterial blood at rest were within the normal range. The level of KL-6 was 1160 ± 1104 U/mL, while the SP-D level was 186 ± 107 ng/mL. Eight patients (17.4%) developed lung cancer after the original diagnosis, and 16 patients already had lung cancer at the time of diagnosis. Squamous cell carcinoma was the most common type (42%), and adenocarcinoma was the second (33%).

The pulmonary function tests (PFTs) are described in Table 2. In spite of the fact that all patients had emphysema and fibrosis on the HRCT, the result showed that only the %DLco decreased significantly among the PFT parameters, a finding which is in agreement with several other studies.

### 3.3. Comparison between CPFE Patients without Lung Cancer and with Lung Cancer at Diagnosis

To analyze the influences of lung cancer on clinical presentation and pulmonary function test, we classified these 46 patients into two groups, a group of CPFE patients without lung cancer (CPFE without CA) and that of CPFE patients with lung cancer (CPFE with CA) at diagnosis. The clinical status and laboratory findings, PFT, and CPI of these patients are summarized in Table 3. There was no significant difference in age and the level of serum SP-D, %VC, %FVC, %FEV_1.0_, FEV_1.0_/FVC (%), %DLco, and CPI between the CPFE without CA and CPFE with CA groups; however, %FRC and %RV/TLC were significantly lower in the CPFE without CA group. On the other hand, the level of serum KL-6 was significantly higher in the CPFE without CA group. Smoking history (pack years) was higher in CPFE with CA group without statistic difference.

### 3.4. Correlation of the Pulmonary Function Tests with KL-6 and SP-D

**Figure 2:**
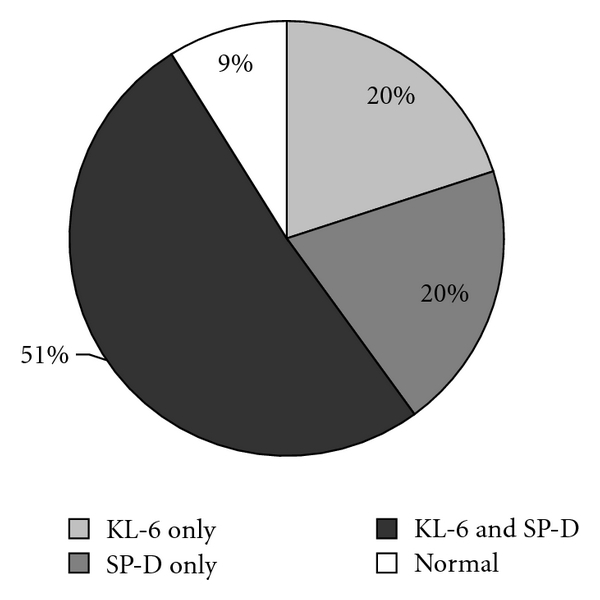
Ratio of patients with high serum levels of KL-6 and SP-D.

Among these patients, 20% had a high KL-6 value, 20% had a high SP-D value, and 51% had both. 91% of the patients had high biomarker values for pulmonary fibrosis (Figure 2). There was a significant negative correlation between KL-6 and %VC (*r* = −0.34, *P* = 0.028), %FRC (*r* = −0.40, *P* = 0.03), %RV (*r* = −0.56, *P* = 0.001), %TLC (*r* = −0.53, *P* = 0.002), %RV/TLC (*r* = −0.36, *P* = 0.03), and %DLco (*r* = −0.33, *P* = 0.036) (Table 4).

There was a significantly negative correlation between SP-D and %VC (*r* = −0.33, *P* = 0.03) and a significant correlation between SP-D and FEV_1.0_/FVC (%) (*r* = 0.34, *P* = 0.03). In a similar manner, the combined product of KL-6 and SP-D (KL-6xSP-D) was also examined. There was a significant correlation between KL-6xSP-D and FEV_1.0_/FVC (%) (*r* = 0.4, *P* = 0.01) and a significant negative correlation between KL-6xSP-D and %VC (*r* = −0.43, *P* = 0.005), %FRC (*r* = −0.41, *P* = 0.01), %RV (*r* = −0.48, *P* = 0.004), %TLC (*r* = −0.6, *P* < 0.001), or %DLco (*r* = −0.32, *P* = 0.046). There was a trend toward an inverse correlation between KL-6xSP-D and %RV/TLC (*r* = −0.32, *P* = 0.06) (Table 4). We used the CPI to evaluate the extent of the pulmonary fibrosis in the patients with CPFE. There were significant correlations between CPI and KL-6 (*r* = 0.461, *P* = 0.005), SP-D (*r* = 0.337, *P* = 0.04), and KL-6xSP-D (*r* = 0.562, *P* < 0.001) (Table 5).

## 4. Discussion

In this study, the importance of both of the biomarkers SP-D and KL-6 is demonstrated, and the product of KL-6xSP-D is shown to be a good indicator for an estimation of the degree of fibrosis in CPFE, a result which is in close correlation with the CPI.

We checked the clinical characteristics of the 46 patients with CPFE who visited our hospital and analyzed them to determine whether KL-6 and SP-D are useful biomarkers for CPFE, as they are for IPF. The prevalence of lung cancer in patients with CPFE was quite high (52.2%) in our study, and squamous cell carcinoma was the most common histological type. Kitaguchi et al. retrospectively reviewed the record of 47 patients with CPFE and found that 22 of those patients (46.8%) had lung cancer. Squamous cell carcinoma was also the most common histological type in their study [4]. The prevalence of CPFE in patients with lung cancer was reported by Usui et al., who found 101 CPFE cases (8.9%) out of 1143 lung cancer patients [11]. All of the patients with CPFE were current or former smokers, and most of them were male. Smoking accounted for the high prevalence of lung cancer in these patients with CPFE, but some genetic susceptibility to chronic smoking-induced inflammation might be involved in the association of CPFE with lung cancer. To take one example of such a factor, a genome-wide association study identified an association between a common variant of the telomerase-related TERT gene and susceptibility to IPF [12].

Shortened telomeres are a risk factor for IPF and are associated with COPD and lung cancer [13–15]. Further studies are required to elucidate whether CPFE is an independent risk factor for lung cancer. Similarly to other studies, only %DLco was significantly low among the pulmonary function parameters in our study. In spite of certain HRCT findings, pulmonary functions in patients with CPFE, except for DLco, were preserved [2, 4]. Pulmonary function tests were influenced both by fibrotic lesions and emphysematous lesions. It is likely that the hyperinflation and high compliance of the emphysematous lesions in the CPFE lungs compensate for the volume loss and low compliance of the fibrotic lesions. In contrast, both emphysematous and fibrotic lesions in the CPFE lungs attenuated the DLco progressively and synergistically.

Other study has implied that KL-6 also rises as a tumor marker for lung cancer [16]. The group of CPFE with CA may have been higher-serum KL-6. However, contrary to anticipation, serum KL-6 was significantly lower in CPFE with CA group. This result showed that the complication of lung cancer had only limited influence on KL-6 value in our study. Compared with pulmonary fibrosis, lung cancer is more frequently pointed out by chest X-rays in the medical checkup. As a result, the patients of CPFE with CA might be discovered at an early stage of pulmonary fibrosis.

The serum KL-6 level in the CPFE patients was negatively correlated with %VC, %FRC, %RV, %TLC, %RV/TLC, and %DLco although they were within the normal limit. KL-6 is a high molecular weight, mucin-like glycoprotein expressed by alveolar epithelial and bronchiolar epithelial cells. KL-6 is reported to be highly expressed by regenerative type II pneumocytes in lung sections from patients with interstitial lung disease [8]. Epithelial breakdown might result in a leakage of KL-6 into the circulation, an effect which can be determined with a commercially available ELISA kit. Serum KL-6 was found to be elevated in various interstitial lung diseases including IPF, nonspecific interstitial pneumonia (NSIP), collagen vascular disease-associated interstitial pneumonia (CVD-IP), and drug-induced and radiation-induced pneumonitis. In 21 patients with IPF and 12 patients with CVD-IP, serum KL-6 was significantly elevated compared to healthy controls and patients with bacterial pneumonias [7]. Satoh et al. reported an increased mortality in patients with IIPs, including patients with IPF, with KL-6 levels > 1000 U/mL [17]. Elevations in KL-6 also were associated with NSIP. In fibrotic NSIP, KL-6 was elevated and correlated with the extent of fibrotic change on HRCT [18]. 

The serum SP-D of CPFE patients was negatively correlated with %VC. SP-D and surfactant protein A (SP-A) are water-soluble members of the C-type lesion superfamily and produced in the lung by alveolar epithelial type II cells. They are thought to be important constituents of the innate immunity of the lung [10]. The serum SP-D and SP-A levels are increased in various interstitial lung diseases, probably due to type II pneumocytic hyperplasia and a disturbed epithelial barrier. SP-D and SP-A are useful biomarkers in patients with interstitial lung diseases [19]. Takahashi et al. showed that the SP-D concentration was related to both the extent of parenchymal collapse and the rate of deterioration per year in pulmonary function [20].

The composite physiologic index (CPI) correlates with the extent of fibrotic change on CT scan more closely and is better linked to mortality than individual pulmonary function indices [21]. Wells et al. constructed a CPI for the morphologic severity of disease to quantitatively calibrate pulmonary fibrosis using pulmonary function tests [6]. The CPI was derived in 106 patients with a clinical and CT diagnosis of IPF. The CPI accounts for coexisting emphysema, which exerts a major confounding influence on pulmonary function indices. The CPI was also used in the analysis performed in the IFIGENIA study [22]. This analysis revealed that patients with a low CPI had a better prognosis.

The combined product of KL-6 and SP-D (KL-6xSP-D) was more closely correlated with the CPI than KL-6 or SP-D alone. KL-6 and SP-D are both biomarkers of pulmonary fibrosis, but a dissociation of these biomarkers is frequently observed. We recently showed that KL-6 was significantly higher in symptomatic than asymptomatic IPF patients, even though the SP-D level was elevated in each group [23]. The difference in the relative level and distribution pattern of KL-6 and SP-D might explain this dissociation between them. It is generally reported that SP-D is significantly correlated with alveolitis (a reversible change), and KL-6 is significantly correlated with chronic fibrosis (an irreversible change) [20]. Therefore, KL-6xSP-D may more closely reflect the severity of the disease and deterioration in pulmonary function.

In conclusion, it is shown that both KL-6 and SP-D are useful biomarkers of the extent of pulmonary fibrosis in patients with CPFE. The results also reveal that KL-6xSP-D is highly correlated with the CPI, which reflects the progression of pulmonary fibrosis in CPFE patients more than either KL-6 or SP-D by itself.

## Figures and Tables

**Table 1:** Clinical characteristics at diagnosis in CPFE patients.

Subjects, *n*	46
Gender, male/female	42/4
Age, years	67 ± 8.7
Smoker (%)	100
Smoking status, current/former	20/26
Smoking history, pack years	58.8 ± 33.9
PaO_2_ (Torr)	82.3 ± 12.3
PaCO_2_ (Torr)	38.7 ± 2.8
KL-6 (U/mL)	1160 ± 1104
SP-D (ng/mL)	186 ± 107
Lung cancer, *n* (%)	24 (52.2)

**Table 2:** Pulmonary function tests in CPFE patients.

%VC	94.7 ± 16.6
%FVC	94.2 ± 16.7
%FEV_1.0_	96.4 ± 18.4
FEV_1.0_/FVC (%)	78.2 ± 10.5
%RV	95.5 ± 33.2
%TLC	90.7 ± 16.1
%RV/TLC	92.5 ± 24.7
%DLco	67.0 ± 17.0
%DLco/V_A_	66.7 ± 14.8

VC: vital capacity; FVC: forced vital capacity.

FEV_1.0_: forced expiratory volume in one second.

RV: residual volume; TLC: total lung capacity.

DLco: diffusing capacity for carbon monoxide.

V_A_: alveolar volume.

**Table 3:** Age and smoking history, KL-6, SP-D, pulmonary function tests, CPI in CPFE without lung cancer (CPFE without CA), and CPFE with lung cancer (CPFE with CA) at diagnosis.

	CPFE without CA	CPFE with CA	*P* value
Age, years	66.6 ± 8.4	67.3 ± 9.3	0.82
Smoking history, pack years	50.9 ± 21.4	73.6 ± 46.7	0.08
KL-6 (U/mL)	1443 ± 1234	629 ± 384	0.002
SP-D (ng/mL)	199.9 ± 108.7	160.1 ± 98.1	0.23
%VC	94.5 ± 14.7	95.3 ± 19.4	0.87
%FVC	93.9 ± 15.1	94.8 ± 19.9	0.88
%FEV_1.0_	97.0 ± 13.8	95.1 ± 25.1	0.81
FEV_1.0_/FVC (%)	79.6 ± 9.2	75.4 ± 12.1	0.22
%FRC	77.8 ± 20.1	100 ± 32.1	0.02
%RV/TLC	83.8 ± 18.9	108.5 ± 25.3	0.003
%DLco	67.4 ± 14.9	66.4 ± 20.1	0.86
CPI	30.2 ± 12.5	30.5 ± 15.4	0.95

**Table 4:** Correlation of pulmonary function tests with KL-6 and SP-D.

	Spearman rank correlation coefficient		
	KL-6	SP-D	KL-6xSP-D
%VC	−0.34	−0.33	−0.43
	*P* = 0.028	*P* = 0.03	*P* = 0.005
FEV_1.0_/FVC (%)	0.21	0.34	0.40
	*P* = 0.17	*P* = 0.03	*P* = 0.01
%FRC	−0.40	−0.16	−0.41
	*P* = 0.03	*P* = 0.36	*P* = 0.01
%RV	−0.56	−0.09	−0.48
	*P* = 0.001	*P* = 0.58	*P* = 0.004
%TLC	−0.53	−0.29	−0.60
	*P* = 0.002	*P* = 0.09	*P* = 0.0004
%RV/TLC	−0.36	−0.04	−0.32
	*P* = 0.03	*P* = 0.83	*P* = 0.06
%DLco	−0.33	−0.09	−0.32
	*P* = 0.036	*P* = 0.57	*P* = 0.046

KL-6xSP-D: the combined product of KL-6 and SP-D.

**Table 5:** Correlation of composite physiologic index with KL-6 and SP-D.

	Spearman rank correlation coefficient		
	KL-6	SP-D	KL-6xSP-D
CPI	0.461	0.337	0.562
	*P* = 0.005	*P* = 0.04	*P* = 0.0006

CPI: composite physiologic index.

KL-6xSP-D: the combined product of KL-6 and SP-D.
